# Early and late signs that precede dying among older persons in nursing homes: the multidisciplinary team’s perspective

**DOI:** 10.1186/s12877-018-0825-0

**Published:** 2018-06-04

**Authors:** Helene Åvik Persson, Anna Sandgren, Carl-Johan Fürst, Gerd Ahlström, Lina Behm

**Affiliations:** 1 Department of Health Sciences, Faculty of Medicine, Lund University, P.O. Box 157, 221 00 Lund, Sweden; 2 Center for Collaborative Palliative Care, Department of Health and Caring Sciences, Linnaeus University, Växjö, Sweden; 3 The Institute for Palliative Care, Lund University and Region Skåne, 221 00, Lund, Sweden

**Keywords:** Dying, Multidisciplinary team, Nursing home, Older persons, Palliative care, Signs

## Abstract

**Background:**

Nursing home residents in Sweden are old, frail and usually have multiple morbidities which often make dying a prolonged suffering. It has been found that older persons at nursing homes receive far less palliative care than younger persons, partly because it is difficult to identify when the final stage of life begins. The identification may help the staff to enable the older person and their families to participate in planning the care in accordance with their own preferences and values. With this in mind the aim was to explore the experiences of early and late signs preceding dying in older persons in nursing homes from the multidisciplinary team’s perspective.

**Methods:**

The focus group method was used to interview 20 health-care professionals on the basis of semi-structured questions. Four focus groups were conducted at four nursing homes in two counties in southern Sweden. The groups included different professionals such as assistant nurses, registered nurses, occupational therapists, physiotherapists, social workers and unit managers. The analysis was conducted according to the focus group method developed by Kruger and Casey.

**Results:**

The analysis revealed one major theme, from unawareness to obviousness, which illustrates that the participants experienced dying as a happening, not a process, and found it difficult to identify early signs. Even though it was a new way of thinking, several suggestions of early signs were presented. The main category “Going into a bubble” illustrates early signs, which meant that the older person showed signs of wanting to withdraw from the outside world. The main category “The body begins to shut down” illustrates late signs, which meant that the older person showed signs that indicate that the body starts to prepare for death.

**Conclusions:**

This study conveys new knowledge concerning the multidisciplinary team’s collective experience of early and late signs that precede dying. This knowledge can increase the understanding of when a palliative care approach needs to be in place at nursing homes. The use of a palliative care approach in care planning requires consensus in the perception of the dying process of frail older persons.

## Background

The average age of the Swedish population is increasing, and it has been calculated that in 2030 one person in four will be 65 or older [1, 2]. Meanwhile, the Swedish “ageing in place” ideology, whereby older persons should be able to live at home for as long as possible, has led to there being a decreasing number of beds in nursing homes. This has in turn led to a situation where it is only the most frail older persons in our society today that are living in nursing homes [3]. Thus nursing home residents in Sweden are old, frail and usually have multiple chronic diseases, making the nursing home a major arena for the provision of palliative care. However, it has been found that palliative care has not been available for older persons dying from multiple morbidities or “old age” to the same extent as for younger persons, perhaps because it can be more difficult to identify when the final stage of life begins [4–6].

The majority of the older persons living in nursing homes also die there [5, 7, 8]. Dying from old age or a chronic disease is often a prolonged suffering with increasingly impaired function, and it is difficult to identify deterioration that signals a short survival or death [9]. Recognizing that a person is dying is often a difficult and complex process [5, 10] but identification of physical, psychological and other changes may help the staff to enable the older person to participate in planning the care in accordance with their own preferences and values and to prepare themselves and their families [11].

Several studies emphasize that information and preparation for the older person can contribute to less fear, fewer misunderstandings and the exploration of wishes with regard to the process of dying [11–13]. However, studies [6, 11] have shown that older persons are insufficiently informed about the imminence of death, for which reason few of them have the opportunity to express their wishes concerning care for the last phase of life. A qualitative study of chronically ill persons in nursing homes [14] points to the importance of a palliative care approach at an early stage of dying at nursing homes: there is likely to be a better quality of life when the older person’s multi-dimensional needs can be satisfied. The importance of a palliative care approach at an early stage of dying, together with early planning, is confirmed by other studies [15–17].

The complexity of the older person’s multi-morbidity may lead to difficulty in identifying when there is a need to renegotiate the goal of care from a general sense to a palliative care approach [4, 6, 18]. Because of the difficulties involved in identifying signs that precede dying in the older person, staff often delay adequate measures to meet palliative care needs [15, 16]. There are a number of limitations to providing the best possible high-quality care in nursing homes, e.g. lack of a multidisciplinary approach and lack of support from physicians [19, 20]. Collaboration in a multidisciplinary team is essential and constitutes a resource when it comes to identifying signs preceding dying in older persons, because of the multi-dimensional skills [12].

There have been a few studies focusing on identifying dying in older persons in nursing homes [21–23]. Brandt et al. [21] focused on physicians’ experiences of dying among older persons with life expectancy of 6 weeks or less. The results indicated that it was difficult for the physicians to predict dying among the older persons who did not have cancer. However, a study [23] focusing on assistant nurses’ experiences has shown that assistant nurses are able to identify both manifest and subtle signs of dying in general. Another study [22] that also focused on assistant nurses’ experiences but also registered nurses’, showed that these professions were able to identify several signs that precede dying, for example that the older person starts falling and stops taking medicines. To the best of our knowledge, however, no study has been done which distinguishes between early and late signs of dying, nor any which focuses on the multidisciplinary team’s collective experiences of early and late signs. Consequently, the aim of the present study was to explore the experiences of early and late signs preceding dying in older persons in nursing homes from the multidisciplinary team’s perspective. In this study, the multidisciplinary team involves members of several professions working daily with the older person.

## Methods

### Design

A qualitative approach with the focus group method was used in this study. Being exploratory in nature, this method is well-suited to the investigation of new areas/phenomena [24]. It involves group interviews in which the discussions among the participants generate the data and the group interaction plays an important role. A focus group usually consists of 3–12 persons from the target group, a moderator leading the discussion and an assistant moderator [24].

### Setting

The Swedish health system consists of inpatient and outpatient specialist care, primary health care and community care which are largely tax-funded. It is a public system that offers equal access for everyone to healthcare, elderly care and social services. The Elderly Reform [3] which came into effect in 1992 shifted the main responsibility for the health care and social services for older persons living at home or in some form of sheltered housing, transferring it from the county councils to the municipalities. This means that older persons with multimorbidity and thus the greatest need for care have priority when it comes to accommodation with provided assistance. In Sweden, the care setting that provides a homelike atmosphere and offers around-the-clock care is the nursing home. The nursing homes consist of small apartments with their own lease. When the older person is so ill and frail that their care needs cannot be met in the ordinary home, a move to a nursing home comes into question. When an application has been made for a place in a nursing home, the social worker in the municipality decides whether the older person’s need of everyday care is so great that moving to a nursing home is necessary. The staff at the nursing homes have varied education and experience. Some of them are trained nurses or assistant nurses, employed by the municipality; and some have no education at all in gerontology or geriatrics. There are also persons among the staff that have an education in social work, generally unit managers [25].

The participants in this study, i.e. staff in the team working around the older person, were recruited from nursing homes from the major project “Knowledge-Based Palliative Care for Frail Older Persons in Nursing Homes” (*KUPA*), where knowledge-based palliative care was implemented in nursing homes through educational seminars [26]. For this study, four out of a total of 30 eligible nursing homes were selected to ensure variation. These four nursing homes were from four municipalities in southern Sweden. They differed in size and represented both rural and urban areas. The interviews were conducted before the staff received the educational intervention concerning palliative care [26].

### Sampling and participants

The unit managers at the four selected nursing homes were asked if they were willing for their unit to participate in this study and all four gave their consent. The unit managers asked the staff whether they wanted to participate in one focus group interview. It had been recommended that they ask a mixture of staff (assistant nurse, registered nurse, occupational therapist, physiotherapist, social worker) that worked daily with the older person and that had at least two years’ work experience in nursing homes. Also, a variation in terms of age, gender and work experience of the participants was desirable. Twenty persons agreed to participate. Their characteristics are shown in Table 1.

**Table 1:** Characteristics of the participants in the study group

	Total study group *N* = 20	Focus group 1 (*n* = 5)	Focus group 2 (*n* = 4)	Focus group 3 (*n* = 5)	Focus group 4 (*n* = 6)
Sex					
Female	18	4	4	4	6
Male	2	1	–	1	–
Age, mean (range)	47 (26–66)	48 (29–59)	46 (35–66)	49 (30–58)	45 (26–66)
Profession					
Assistant nurse	5	1	1	2	1
Nurse	3	–	1	1	1
Occupational therapist	4	1	1	1	1
Physiotherapist	4	1	1	1	1
Social worker	2	1	–	–	1
Unit manager	2	1	–	–	1
Work experience current workplace, mean (range), in years	7.5 (1–18)	5.8 (1–15)	9 (5–14)	9.6 (1–18)	5.6 (1–12)
Previous experience of caring for seriously ill and dying persons, no. persons					
Geriatric unit	2	–	1	–	1
Home health care	4	1	1	1	1
Nursing home	12	3	2	3	4
Palliative unit	1	–	–	1	–
Other	1	1	–	–	–

### Data collection

Each unit manager decided time and place for the focus group interview. The interview guide consists of semi-structured questions and the interview was done in a detached room at the nursing home. The participants were asked to fill in a questionnaire before the interviews started with background information (shown in Table 1). The focus group session started with everyone in the group presenting themselves, with name and profession. The interview guide developed for this study included the following main questions: What would you say are early signs that can be identified in an older person that has a palliative care need? What would you say are late signs that can be identified in an older person that has a palliative care need? To further deepen the discussion, probing questions were used, such as: What do you think about that? Can you tell us more? Is there anything else? In this study, the term “a palliative care need” means that the older person has physical, psychological and social needs related to the fact that the goal of care has shifted from being curative to being focused on the prevention and relief of suffering. The meaning of “early signs” (i.e. signs from months up to a year before dying) and of “late signs” (i.e. signs in the end of life — days or weeks before dying) was explained to the participants before the interview began.

The focus groups were led by a moderator (first author, H.Å-P) and an assistant moderator (last author, L.B). According to Krueger and Casey [24] the task of the moderator is to lead the discussion, keep it on-stream and listen to the participants, whilst that of the assistant moderator is to take notes, ask additional questions and handle the digital recorder and the logistics. During the interviews the moderator and the assistant moderator worked actively to ensure that all the participants were able to express their views about the subject. The four focus group interviews were conducted in Swedish and lasted 48–83 min. The digitally recorded interviews were transcribed verbatim.

### Data analysis

The analysis was conducted according to the focus group method developed by Krueger and Casey [24]. The transcripts were read through several times in order to create an overall picture of the data and to gain a deeper understanding of the team’s experiences of identifying early and late signs preceding dying. Thereafter, meaning units were identified in relation to the aim of the study. A meaning unit derives from a discussion among staff concerning one issue and comprises one or more sentences [24]. The meaning units were then condensed in order to shorten the text but still maintain the content. Finally, the meaning units were labelled with codes, and views with similar meanings were gathered in sub-categories, main categories and later a theme.

The first author (H.Å-P) conducted the analysis together with the last author (L.B), who also did a parallel independent analysis concerning reading the transcripts and extracting meaning units, codes and categories. Rigour or trustworthiness according to Krueger and Casey [24] was upheld by means of investigator triangulation. Regular meetings were held throughout the analytical process where the other authors were involved in reading the interviews (A.S, G.A) and reflecting on the content of the different concepts in the analysis (A.S, C-J. F, G.A). This process lasted until agreement was reached.

## Results

The results consist of one theme, two main categories and eleven sub-categories. The theme “From unawareness to obviousness” describes the overall manner in which the participants identify early and late signs preceding dying among older persons in nursing homes (see Table 2).

**Table 2:** The theme, main categories and sub-categories

From unawareness to obviousness	
Going into a bubble	The body begins to shut down
Lack of interest in the surrounding world Low mood Increased sleep Newly added confusion Reduced physical ability Decreased appetite	Reduced circulation Increasing worry and anxiety Stopped eating and drinking Loss of consciousness Changed breathing pattern

### From unawareness to obviousness

It was clear from the interviews that dying was seen as a happening and not a process, which meant that dying was associated with end-of-life. Early signs were not identified in practice, which of course made it difficult for the participants to answer the question about how they identified them, while late signs were well-known and clear. Even though it was a new way of thinking, several suggestions as to early signs preceding dying were described. The main category “Going into a bubble” accounts for how early signs preceding dying in older persons were described, and the main category “The body begins to shut down” accounts for how late signs were described.

### Going into a bubble

The overall image of early signs preceding dying in older persons was that of going into a bubble. The participants described it as being a question of the older person’s showing signs of wanting to withdraw from the outside world and not caring about things to the same extent as before. The early signs described were multidimensional — physical, psychological, social and existential — and were experienced as small and subtle, which required a sharp eye or a person that knew the older person well to notice them. The main category *Going into a bubble* can be explained by way of the six sub-categories: *Lack of interest in the surrounding world, Low mood, Increased sleep, Newly added confusion, Reduced physical ability* and *Decreased appetite*.

#### Lack of interest in the surrounding world

A common early sign preceding dying was that the older person lost interest in the surrounding world, shielding themselves from it in different ways and usually beginning to talk about past experiences in their lives, e.g. childhood. One example was an older person who previously had been social but no longer appreciated visits or preferred fewer visits, another example was an older person who previously had looked forward to visits by children and grandchildren but no longer did so. Some older persons start to prefer to have their food in their room, some become more silent and want to be left alone. Or there can be less interest in such activities as exercise, watching television and listening to the radio.

*Physiotherapist (PT): You’ve met a few... who’ve died, after you’ve got to know them over time. Then if you’ve looked back... — well, I can see something I’d never given much thought before.... A loss of interest, sort of, in sport, in sports results... Well well...*

*Assistant nurse (AN): Yes, I know just what you mean... When it comes to watching TV... or perhaps listening to music...*

*PT: Yes. It’s not so interesting...*

*Registered nurse (RN): Yes, and you hear “I don’t want to watch TV. I haven’t got the energy.”*

*PT: I wouldn’t say they haven’t got the energy, the ones I have in mind. They’re just not interested.... There was one, for instance, who went to football a lot — active and watched football... and then later on didn’t have much interest in the results.*

#### Low mood

One possible early sign preceding dying was a change in the older person’s mood, which could become depressed, irritable, low. This change could result in the older person’s not caring about being involved to the same extent as before in (to take one example) decisions affecting their everyday life — and some leave the responsibility entirely to the staff. The low mood could manifest as anything from saying it is not fun getting old to expressing a desire not to live any more.

*RN: They’re quieter.*

*Moderator (MA): Mmm...*

*AN1: Depressed.*

*AN2: Shut yourself up in a world of your own.*

*MA: In what way do you think those are early signs, then? Shutting yourself up, becoming depressed and quiet... — why is that an early sign, do you think?*

*AN1: Well, you maybe spend a lot of time thinking about things... when you know what is going to happen... — and of course that can be very tough.*

*MA: Mmm...*

*AN1: Then anyway there may be times when you don’t feel up to talking to anybody, and you shut yourself up.*

#### Increased sleep

Increased sleep was discussed as an early sign. There could be an increased desire to lie down and rest, for example wanting to lie on the bed again and rest after the morning routine. Also it could become difficult to wake the older person up, either in the morning or during the rest of the day. Furthermore the older person might fall asleep more often during the day, while sitting, for instance, in the dining-room or in an armchair. Or an older person who has never before taken a rest after dinner suddenly feels a need to do so.

*AN: So it can be someone, say, that’s never had a lie-down after dinner before but has one now….*

*RN: Mmm...*

*AN: Oh, but yes, it’s a bit that way... and we look at it as more or less normal* [laughs]*. I mean, if you’re over 80, 85... your body’s tired.*

*RN: Yes.*

#### Newly added confusion

Confusion was seen as an early sign preceding dying and could be observed as the older person’s suddenly beginning to behave and express themselves in a different way than before. The change was seen as a sign of something not being right. The participants were in agreement that any sign of confusion always should be carefully investigated and that different possible causes always must be excluded before it could be interpreted as a sign preceding dying.

*Unit manager (UM): Yes, I’ve come across confusion.... It’s often spoken of, but you need to look into everything else first. If a person’s confused it can be due to any of the things we’ve talked about, from an infection of the urinary tract to the effect of medicines.*

*Occupational therapist (OT): Mmm...*

*UM: So there are such a lot of things that can lie behind confusion... But I think that’s a sign too.*

*MA: Mmm...*

*Social worker (SW): And of course a change of environment, too, is... causes confusion.*

*AN: Or, in the same sort of way, if they’re confused they perhaps don’t know how to use their fork when they’re going to eat.*

#### Reduced physical ability

The fact that the older person becomes weaker and has a greater tendency to fall was discussed and could be seen as an early sign preceding dying. It was also brought up that a change in the ability to perform daily activities because of a general decline in function could be a sign. This general decline could be manifested in the form of a small change in a person’s pattern of movement. Perhaps a need of help had arisen, where for instance a person had lost the ability to stand up without the aid of a mobility device.

*MA: Well, the next question that comes up is: Is there anything more you can say about these early signs?*

*AM: Mmm... Falling, you mentioned...*

*OT: Mmm...*

*AM: Is it something you notice? ... Do you get sent for then?*

*PT: Yes, when there’s a fall that’s out of the ordinary.... Yes, and you often see that, well, somebody that hasn’t fallen at all before starts falling, and then usually soon after they get “doddery”.*

*MA: Yes.*

*PT: But then you realize that this was the beginning of the end.*

#### Decreased appetite

Decreased appetite could be seen as an early sign. The older person ate much less, and the fact that the person’s clothes became too large confirmed that there had indeed been a loss of weight. The participants believed that sometimes the older person had decided to reduce their food intake because they did not want to live any more. When an older person refused food that they previously enjoyed it could be seen as a resignation.

*AN: Tired, stop eating….*

*OT: Yes, getting tired and stopping eating… that’s the sort of thing we see….*

*AN: And of course that’s what we notice first… They may drink but….*

*OT: Yes….*

*AN: But not as much and….*

*AM: But is that an early sign?*

*AN: That they stop eating — yes, I think so. You see, all too often they’ve already... — the thing is, some of them have simply decided “I don’t want to carry on any more” and the only thing they can do about it is to stop eating.*

*OT: Mmm...*

*AN: And that’s what we see so many times... that they don’t want to carry on any longer.*

### The body begins to shut down

The overall impression of late signs preceding dying was that the body begins to shut down, by which is meant that the older person shows signs that indicate that the body is starting to prepare for death. The late signs could be both physical and psychological and were seen as indicating that the older person was in end-of-life. The participants could clearly account for late signs and talk about them without vagueness. It became fully evident in the discussion that dying was something familiar which occurred in the participants’ everyday work and that end-of-life care was something the participants had experienced several times. The main category *The body begins to shut down* can be explained by means of five sub-categories: *Reduced circulation, Increasing worry and anxiety, Stopped eating and drinking, Loss of consciousness* and *Changed breathing pattern* (see Table 2).

#### Reduced circulation

Reduced circulation was described as a late sign of dying. The participants could observe changes in the colour of the older person’s skin and that they were especially observant of the hands and feet, which could be marbled. Furthermore, the participants pointed out that they were observant regarding cold hands and feet because that indicates that the blood circulation is starting to fade, which is a sign related to the body’s beginning to shut down. If pressure ulcers occur, this could be seen as a late sign of dying because the older person often becomes bedridden at that stage and the skin often becomes red and affected.

*RN: Because I think otherwise there’s so much lying in bed, pressure ulcers won’t be long coming.*

*AN: Nowadays things are so good... the mattresses and that. We don’t need to put in the work we used to.*

*UM: I was just about to say, I think this place is pretty good in that way. Worse at the hospital….*

*OT: Yes, I was going to say that a lot come from the hospital with ulcers, unfortunately. But we’re pretty good at keeping it in check here ….*

#### Increasing worry and anxiety

A late sign of dying mentioned by the participants was anxiety, which could manifest as anger and frustration. It was brought up for discussion that as older persons are often less able to talk at this stage, they try to express themselves through body language, e.g. waving their arms or making a noise. Another late sign which was often seen was that the older person had a broader worry which manifested as restlessness, incoherent talk and hallucinations.

*SW: Can’t it be that the brain begins to sort of go into decline — the brain’s activity, that is — and you see things, and become a bit….*

*RN: Hallucinations and that sort of thing?*

*SW: Yes, exactly.*

*RN: Maybe so. At the same time there are pretty big differences, I’d say, when it comes to starting to hallucinate. There may be some people who never hallucinate at all, but it’s a pretty common sign, I suppose...*

*AN: I hadn’t thought about it before….*

#### Stopped eating and drinking

In the end-of-life, the older persons stopped eating and drinking, which according to the participants is a late sign of dying. The fluid intake is usually sparing, with the result that the medicine cannot be taken orally and injections and mouth care are called for. The participants were surprised at how long an older person can survive with hardly any fluid but were unanimous in thinking that the person does not seems to suffer without food or fluid.

*AN: Then perhaps they don’t eat anything anymore, and drink hardly anything at all. Well, then it’s good care that’s called for, and seeing that the person isn’t in pain or in a state of anxiety.*

*RN: Yes, it’s more a question of their being in pain. No desire to eat — the body can’t receive food. It goes downhill very fast in the final phase.*

*AN: Yes. Can’t take their medicines….*

*RN: Well, in the end it’ll be injections instead.*

*AN: Exactly.*

#### Loss of consciousness

The participants pointed out that in the end-of-life the older person begins to sleep more and more and finally loses consciousness; and when this happens, it is a clear late sign preceding dying. This sign was for many of the participants a reminder that this is when end-of-life care actually takes shape. Once an older person became bedridden, it was not long before the person’s consciousness began to deteriorate, leading to unconsciousness. At this stage, when the older person is not contactable, the deterioration which led to death often went relatively fast.

*MA: What signs of deterioration do you see appearing in an older person in life’s final phase?*

*AN: Perhaps there’s less and less contact….*

*MA: Mmm...*

*AN: Sleeping more, beginning to feel anxiety and... — now I can see in front of my eyes, now that you ask me, a person lying in bed and having next to no energy, where it’s a question of days...*

*AN, OT: Mmm….*

*AN: Or perhaps a week or so...*

#### Changed breathing pattern

Another sign that the participants observed during the last days preceding dying was that the breathing pattern changed. The older person’s breathing might become slower, irregular, shallow and wheezy. Moreover, it was mentioned that the older person could hold their breath for a long time, which was described as frustrating by the participants because every breath was believed to be the last. This process could carry on for a while until death occurred.

*PT: Breathing, sometimes it can change, of course. And you think, “Oh hell, is something going on with their breathing?” Then you hear it starting to compensate in some way, turning into wheezing.*

*AN: Mmm... yes, there can be wheezing and long pauses in breathing sometimes, and you think the end has come. But it can go on like that, that much we know. If there’s no one keeping vigil you go by the sound of their breathing and then you probably need to be there even more often.*

*MA: Mmm...*

## Discussion

This study shows that the multidisciplinary teams working with older persons in nursing homes found it difficult to identify early signs that precede dying. Whilst at the beginning of the interviews the staff found it hard to imagine what such signs might be, they later — with the help of the discussions in the focus group — started to get closer to several signs which could be considered early. One reason why the staff found it hard to identify early signs might be that they were not used to seeing dying as a process which extends over a time-period. The early signs were described as subtle and were sometimes seen as both signs that precede dying and signs of something else than dying such as a disease. Porock and Oliver [22] raise one possible reason why early signs preceding dying get little attention at the nursing homes: talking about death is taboo among the staff, for which reason an increasing awareness that the older person is going to die can be hard to handle. An earlier study by Sahlberg-Blom and colleagues [23] on assistant nurses’ experiences of signs of dying found signs similar to those we ourselves found. The signs presented in their study cover physical and psychosocial changes in older persons and include both subtle signs of dying such as feeling a desire to die and manifest signs such as body changes, for example fatigue and difficulty in breathing. Another study [27] which explored how nursing home staff (nurses, assistant nurses and social workers) managed the transition from routine care to end-of-life care found that the staff discussed physical changes as a core aspect of the transition. In contrast to those results, the early signs identified in our study were both physical, psychological and social in nature, i.e. the person was seen as a whole. The reason for the differences in the results might be that the staff were of different professions and thus the groups had a multidimensional view of the older person.

One early sign mentioned by the staff was that the older person felt a greater need to go through their life from childhood to the present and talk about past experiences. One way to explain this result is by means of the theory of gerotranscendence developed by Tornstam [25, 27, 28]. This theory about the ageing process states that human progress is a life-long development that stretches into old age and finally results in a new understanding of life. One of the levels in gerotranscendence is the cosmic level, which includes the dimensions of time and space. Changes occur in the perception of time and space which can cause the border between present and past to become blurred and involve a return to and reinterpretation of childhood. With application of the theory of gerotranscendence the fact that the older person shows signs of wanting to withdraw from the outside world and of not caring about things to the same extent as before can be interpreted as a natural progression towards maturation and wisdom, instead of it as disengagement or apathetic behavior. Wadensten [29] claims that the theory of gerotranscendence can improve our knowledge of the transition into old age and provide a basis for staff’s discussion of how to provide optimum care for the older person and how to support ageing. If the staff at nursing homes had knowledge about gerotranscendence, it would increase their understanding of the older person’s needs, perhaps (to mention but one possible benefit) providing the initiative for more talks. Guidelines for nursing have been developed from the theory of gerotranscendence [30], and these could be used as a tool for the staff to support the older persons in their progress towards gerotranscendence. In addition an intervention has been made to introduce the guidelines to staff in nursing home, involving eight occasions with lectures and discussion in groups [31]. The results showed that many of the staff had a different view of the signs of gerotranscendence after the intervention and experienced them now as a normal part of ageing instead of as pathological.

A recurring early sign was resignation: the older person sometimes seemed to have given up and did not want to live any more. This resignation could appear in different forms among the older persons, e.g. withdrawal from social contexts, decreased appetite and lack of motivation. Montoya-Juarez and colleagues [32] state that persons use psychological defences to cope with the challenges that arise in the end-of-life. Resignation can be seen as one such defence and can be shown through negative feelings and thoughts which are given verbal expression. In addition, resignation can also include a feeling of acceptance which may provide a certain amount of calm before death occurs. Resignation and dejection have been raised in other contexts. In a study by Tollén, Fredriksson and Kamwendo [33] the older persons were still relatively independent, but when they started experiencing impaired function there arose feelings like emptiness, resignation and dejection. Even if they knew that they should try to engage in different activities, they did not take the initiative. Resignation can also be seen as a part of gerotranscendence [25, 27, 28]. According to the theory the fear of death has decreased and the older person can talk in greater depth about dying and express such feelings as that they do not want to live any more.

In contrast to the early signs that precede dying, late signs of dying were familiar. The staff had the knowledge concerning which signs to look for and they used it in everyday practice. It was clear from the interviews that dying was seen as a happening rather than as a process, meaning that it was restricted to the last days or weeks of the older person’s life. These results are in line with results obtained by Beck and colleagues [34] which showed that assistant nurses in nursing homes experience palliative care as lasting only for a short and limited time. The focus on late signs might be attributable to the fact these signs are obvious, i.e. they are familiar, clear and prominent in the last days or weeks. The late signs are also similar to those that have been described in the literature [21–23] and the staff are well aware of them. In contrast to the holistic view of the older person connected to early signs preceding dying, the participants mainly highlight the physical and psychological aspects of late signs. This is in line with earlier research [35, 36]. This might be explained by the fact that during the last week/days the older person is often bedridden and unconscious. However, in order to provide a holistic care and abide by the basic values of palliative care (presence, wholeness, knowledge and empathy), social and existential aspects also need to be taken into consideration [18]. Even though the older person is unconscious, the right to be treated with dignity remains, and it is regulated in the Swedish Health and Medical Services Act [37].

Today, person-centred care is applied in nursing homes [38–40]. However, an early preparation for end-of life seems to be lacking. Waldrop and colleagues [41] argue that all nursing home residents are admitted because of a medical crisis which has necessitated institutionalized long-term care and that this fact implies that all nursing home residents are to be considered as dying, although not necessarily imminently. Thus a palliative care approach could be put into place from the very start of a person’s residence at a nursing home, e.g. using advanced care planning. Advanced care planning is an early ongoing communication and decision-making process with the older person and their next of kin which addresses the approaching death. Studies show that advanced care planning can improve the quality of end-of-life care (EoLC) [42], increase the number of EoLC discussions and enhance concordance between patient preferences and provided care [43, 44]. However, a recent review highlights that implementation of advanced care planning in nursing homes requires the involvement and education of staff, including nurses, physicians and leaders [45].

There are some methodological issues that need to be discussed, first the recruitment process. We asked the unit managers of the nursing homes to ask the staff if there was anyone interested in participating in the study. This approach in recruitment can be seen in two ways. There can be a methodological problem with regard to volunteering because a request from the unit manager to participate in a study can be perceived as mandatory. However, Kreuger and Casey [24] indicate that a person may see it as a good thing to be chosen by the unit manager: the person feels honored and special, and participation is experienced as something positive. The unit managers included the staff who had a special interest in palliative care and those who thought the study seemed interesting. The researcher’s perception with regard to the focus groups was that everyone had decided for themselves whether they wanted to participate or not and that everyone was engaged and interested, which led to a comfortable climate during the interviews.

Another issue that needs attention is the representation in each focus group of different professions from the multidisciplinary teams at the nursing homes. The goal was that professions like assistant nurse, registered nurse, occupational therapist, physiotherapist, social worker and unit manager should participate in each interview, but it was not possible to obtain all professions for all groups. However, the results of this study can be seen as a whole, which could make the drop-out less likely to influence the results remarkably. One profession which was represented in all focus groups was that of assistant nurse. Studies [23, 34] show that the assistant nurses’ practical knowledge is of great importance for the multidisciplinary team. This was also evident in the interviews. In relation to this issue, one might question the inclusion of other professions in the study. The registered nurse and the assistant nurse are the ones who are working closest to the older person and might therefore be the ones with most knowledge of signs that precede dying. This is also evident in earlier studies [22, 23]. However, as the complex needs of frail older persons require diverse professionals to be able to offer a holistic care [13], the experience represented by the multi-professional team is of importance. One profession that is a natural part of the team but not included in our focus groups is that of the physician. The decision to exclude physicians was based on the fact that physicians in Swedish nursing homes are employed by another organization and only come as consultants, i.e. do not work daily with the older persons.

Within the focus groups most of the staff knew each other before the interviews, which might have affected the answers and the discussion. The staff felt calm and secure in the interviews because there were persons they know, which could have meant a more comfortable climate and a greater readiness to discuss sensitive matters. Reflecting our own experiences, Kreuger and Casey [24] argue that there must be a concern in focus group interviews to reach a balance whereby there is enough variation within the group at the same time as this variation is not such that some of the participants become silent because the other participants have greater education or experience. In the focus group interviews the moderator and the assistant moderator worked actively to ensure that all staff should be able to speak.

The study includes four nursing homes in two counties in four municipalities in southern Sweden, which can be seen as a narrow sample. However, in qualitative studies generalization is not the goal, which is instead to present results which can be transferred to similar contexts. Whether the results are transferable to another context is a question for the reader’s assessment [46].

## Conclusions

The team working with the older person found it difficult to identify early signs that precede dying mainly because they did not see dying as a process but as a happening, restricted to the last weeks or days of the older person’s life. One early sign that the participants identified in different ways among the older persons was resignation, e.g. withdrawal from social contexts, lack of motivation and low mood. Another sign was that the older person developed a need to go through their life, from childhood to the present. Late signs that precede dying were familiar, observed by the staff in everyday practice. The team’s collective experience of early and late signs that precede dying constitutes new knowledge that has never been shown in the literature before. This knowledge can increase the understanding of when a palliative care approach needs to be in place at nursing homes. This approach is relevant to advanced care planning. It would prepare both the staff and the older person for dying and make it possible to conduct person-centered care. Knowledge of ageing, frailty and the dying process in older persons needs to be part of the staff training. Also joint discussions within the organization regarding the preparedness for palliative care might encourage the managers to facilitate the implementation of a palliative care approach in nursing homes.

## Abbreviations

AM: Assistant moderator
AN: Assistant nurse
EoLC: End-of-life care
MA: Moderator
OT: Occupational therapist
PT: Physiotherapist
RN: Registered nurse
SW: Social worker
UM: Unit manager
